# Sagittal and Vertical Changes After Aligner Versus Acrylic Splint Expansion in Mixed Dentition: A Prospective Trial

**DOI:** 10.1016/j.identj.2026.109579

**Published:** 2026-04-27

**Authors:** Lanxin Lu, Lingling Zhang, Chengri Li, Lei Lei, Yanqin Lu

**Affiliations:** a Department of Orthodontics, Xiangya Stomatological Hospital and Xiangya School of Stomatology, Hunan Engineering Research Center for Oral Digital Intelligence and Personalized Medicine, Hunan Key Laboratory of Oral Health Research, Central South University, Changsha, China; b Department of Dermatology & National Engineering Research Center of Personalized Diagnostic and Therapeutic Technology, Xiangya Hospital, Central South University, Changsha, China

**Keywords:** Invisalign First aligners, Cephalometrics; Mixed dentition; Arch expansion

## Abstract

**Introduction and aims:**

The efficacy of acrylic splint expander (Splint) and Invisalign First aligners (First) in increasing transverse width in mixed dentition have been well documented, but little is known about their sagittal and vertical effects. Since sagittal and vertical changes are particularly critical in growing patients, the objective of this study was to assess and compare the sagittal and vertical treatment effects of First and Splint in mixed dentition with transverse maxillary deficiency.

**Methods:**

Sixty subjects were included after propensity score matching (PSM) from initial 163 cohort: the Splint group (n = 20), the First group (n = 20), and the Natural Growth (Natural) group (n = 20). Lateral cephalograms were acquired before the initiation of treatment (T0) and at a minimum of one year after treatment (T1). Sixteen cephalometric variables were assessed to detect differences within and between groups from T0 to T1. Intra-group analyses were conducted using paired *t*-tests, while inter-group comparisons were performed using analysis of variance (ANOVA). Statistical significance was set at *P* < .05.

**Results:**

In all groups, a significant increase (*P* < .05) were observed in ANS-Me, N-Me, and S-Go during the T1-T0 period. In the Splint group, ANB and NA-PogA significantly decreased (*P* < .05), whereas SNB significantly increased (*P* < .05). In the First group, both overjet and overbite significantly decreased (*P* < .05), with a greater reduction observed compared to the other two groups (*P* < .05). No significant differences were found in the remaining variables, either within or between the groups.

**Conclusions:**

Splint therapy improved skeletal relationships, resulting in reduced facial convexity. First therapy produced a pronounced reduction in overjet and overbite primarily through dentoalveolar effects.

## Introduction

Maxillary transverse deficiency (MTD) is a common skeletal irregularity encountered in orthodontics during the mixed dentition which could hardly self-correct since maxillary development in the transverse dimension stops early.^1^ Untreated MTD is associated with the development of severe malocclusion, including Class II relationships and midfacial deficiency. In addition to occlusal consequences such as abnormal tooth eruption, it may also lead to significant respiratory problems and interfere with normal craniofacial growth and development.^2^, ^3^, ^4^ Therefore, correcting MTD in growing patients is of great importance to promote normal maxillary growth and improve sagittal and vertical relationships between the maxilla and mandible, thereby contributing to overall craniofacial and general health.^5^

The acrylic splint expander (Splint) is one of the most commonly used appliances for the correction of MTD in mixed dentition.^6^ Beyond increasing transverse width, splint expansion has also been reported to influence sagittal and vertical craniofacial dimensions. Previous studies have noted downward and backward rotation of the mandible following arch expansion protocols,^7^ resulting in increases in vertical facial dimension and mandibular plane inclination.^8^ In addition, downward and forward displacement of the maxilla after splint therapy has also been reported.^9^ To mitigate or minimize excessive downward and anterior displacement, bonded rapid palatal expansion appliances have been recommended.^10^^,^^11^ Conversely, some authors have reported minimal vertical control with splint expanders.^12^ Although previous studies have suggested that acrylic splint expanders are capable of inducing skeletal effects during the mixed dentition stage, inconsistencies in appliance design and study duration have led to conflicting findings. This unresolved issue represents a clinically relevant gap, as the objectives of early orthodontic intervention in growing patients extend beyond dental alignment to include optimization of transverse, vertical, and sagittal craniofacial skeletal relationships.

Aside from the traditional acrylic splint expander, the Invisalign First (First), a new appliance introduced in 2018, is intended for mixed dentition patients with MTD and has revolutionized interceptive orthodontic treatment. As a means of comfort and aesthetics, First has been proved efficient in expanding the maxillary arch during the mixed dentition phase.^13^^,^^14^ Our previous study indicated that it could be a reasonable option for patients with mild to moderate MTD,^15^ which was confirmed by a recent study comparing transverse changes treated with First to Hyrax expander.^16^ However, whether First can produce comparable growth-related sagittal and vertical skeletal effects remains unclear. This uncertainty underscores the need for a more comprehensive and evidence-based evaluation to guide orthodontic clinical decisions.

Sagittal and vertical measurements of children during mixed dentition may vary with growth. For example, a reduction in profile convexity and a forward rotation of the mandible were observed in teenagers from age 9 to 18.^17^^,^^18^ Besides, a mild flattening of the facial profile, characterized by a reduction in the ANB angle, was observed during the pubertal growth period.^2^^,^^19^^,^^17^ In subjects with hyperdivergent patterns, the overbite slightly decreased from 9 to 18 years old.^20^ Nonetheless, previous researches lacked blank control to rule out the impact of growth on sagittal and vertical dimensions during the mixed dentition period. Clinicians could only rely on experiences and a small amount of poor-quality research evidence when formulating therapy strategies. Therefore, we creatively included a natural growth group (Natural) in this study to account for the impact of natural growth thus to get more accurate results.

The scientific validity of clinical research may be affected by many confounding factors. Propensity score matching (PSM) is a statistical method used to reduce bias caused by observed covariates,^21^ which has been extensively used in clinical studies to assess and compare the effectiveness of different treatments.^22^^,^^23^ In this study, the patient's cervical vertebral maturation (CVM), gender, and Angle's classification before treatment were considered as interfering variables which may affect the treatment effect assessment. Thus, we innovatively implemented PSM to equalize the distribution of these confounding variables in the baseline data across groups to obtain high-quality research results.

Hence, the aim of this prospective clinical study was to assess and compare the sagittal and vertical effects of First and Splint during the mixed dentition phase using digital cephalometric measurements, while including a natural growth group to eliminate the impact of growth factors, thus providing reference for orthodontists in developing personalized treatment plans for expansion in growing patients.

## Materials and methods

This prospective cohort study initially recruited 163 patients who regularly visited the Hunan Xiangya Stomatological Hospital, Central South University, between May 2022 and May 2024. This study was registered in the Chinese Clinical Trial Registry on February 1, 2022 (registration number: ChiCTR2200056220).

### Sample size calculation

Based on the data reported by Çörekçi et al,^9^ the standard deviations of the ANB angle were estimated as 0.74 and 1.18. The significance level was set at 0.05 with a statistical power of 95.37%. Sample size estimation indicated that a minimum of 14 subjects per group was required.

### Characteristics of participants

The inclusion criteria were as follows: (1) mixed dentition with complete eruption of first molars; (2) transverse discrepancy between the maxillary and mandibular arches; (3) mild to moderate crowding; and (4) cervical maturity stages 1-3 (CS1-3). The exclusion criteria included: (1) systemic diseases; (2) Class III malocclusion or crossbite; (3) prior orthodontic treatment.

### Propensity score matching

Using PSM method, a one-to-one nearest neighbour matching method was employed. The CVM, gender, and Angle's classification of malocclusion were evaluated and noted as confounders for patients in the First and Splint group. CVM was assessed using pre-treatment lateral cephalometric radiographs and categorized into six stages according to established criteria recommended by previous literature.^24^ CS1-CS2 correspond to the prepubertal stages of skeletal development. The patient's gender and classification of Class I or II malocclusions were recorded as binary variables. A detailed flowchart of the sample inclusion process is provided in Figure S1.

A χ2 test was employed to contrast the initial data for the final treated sample in the First and the Splint group, for balancing confounding factors (Table 1). The Natural group consisted of adolescents aged 7 to 10 who had not received any expansion therapy and were regularly monitored by the researchers during the study period. PSM was performed on the initial cohort of 163 patients, resulting in a final matched sample of 60 patients, with 20 patients per group.

**Table 1:** Baseline data for patients in the First group and Splint group after PSM.

	Baseline data after PSM		*P* value
	First group	Splint group	
Gender			
male	7	10	.34
female	13	10	
Age			
CS1	11	10	.75
CS2	9	10	
Angle’s classification of malocclusion			
Class I	5	6	.72
Class II	15	14	

*P < .05 indicates statistical significance; CS1-2, prepubertal stage of development.

### Treatment protocol

**Figure 1:**
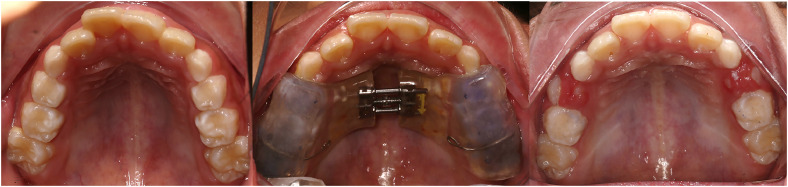
Changes in maxillary arch morphology before and after Splint treatment.

Participants in the Splint group underwent treatment with acrylic splint expanders following the established protocol. As shown in Figure 1, the acrylic splint expander covered the maxillary deciduous canines, first and second deciduous molars, as well as the maxillary permanent first molars. After one week of removable wear for adaptation, the acrylic splint expander was bonded for expansion following a standardized protocol, which involved two turns per day (0.25 mm per turn) until the facial cusps of the maxillary posterior teeth approximated the lingual cusps of the mandibular posterior teeth. The bonded appliance was typically kept in place for retention.

**Figure 2:**
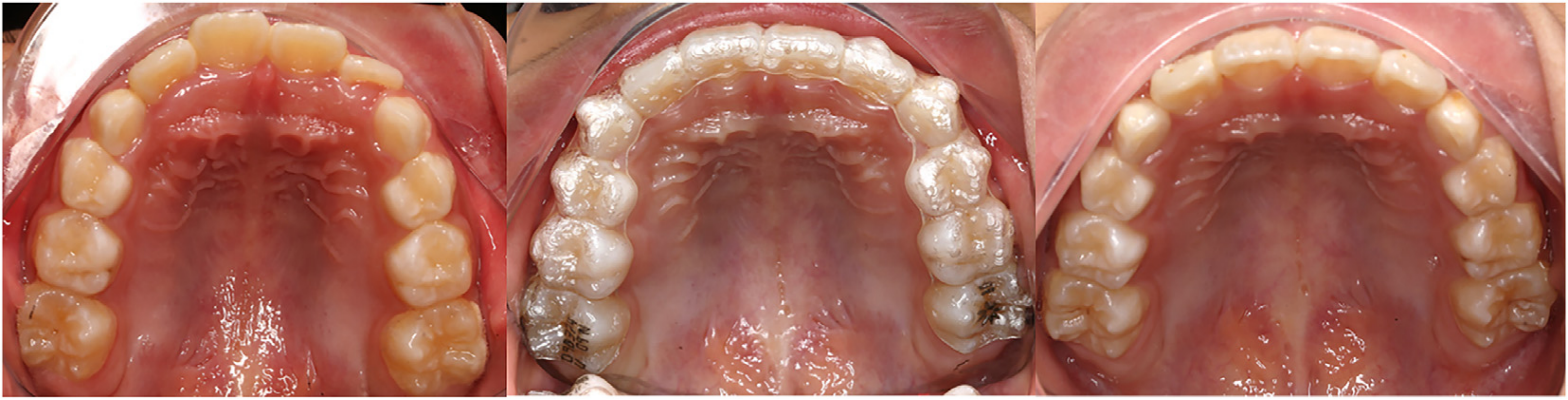
Changes in maxillary arch morphology before and after First treatment.

Patients in the First group were treated with Invisalign First using a standardized ClinCheck expansion protocol (Figure 2). In the First group, ClinCheck plans incorporated a sequential staging expansion protocol in which the molars were expanded first, followed by simultaneous expansion of the primary molars and canines, with 0.25 mm expansion per stage. Patients in the First group were mandated to apply their aligners for the entire day, excluding meal times and oral hygiene routines, and replaced every week. Every two months, the orthodontist verified the aligner position and attachment positions to ensure they were in place. For instances of tooth loss and eruption, patients can obtain a new scan to reconstruct the aligners according to the original prescription. After treatment completion, passive appliances were worn as retainers. For patients treated with clear aligners, treatment compliance was routinely monitored using manufacturer-provided compliance indicators, which offer an objective estimation of daily wear time. In addition, patient compliance at each follow-up visit was documented in the clinical records for all three groups.

### Treatment evaluation

**Figure 3:**
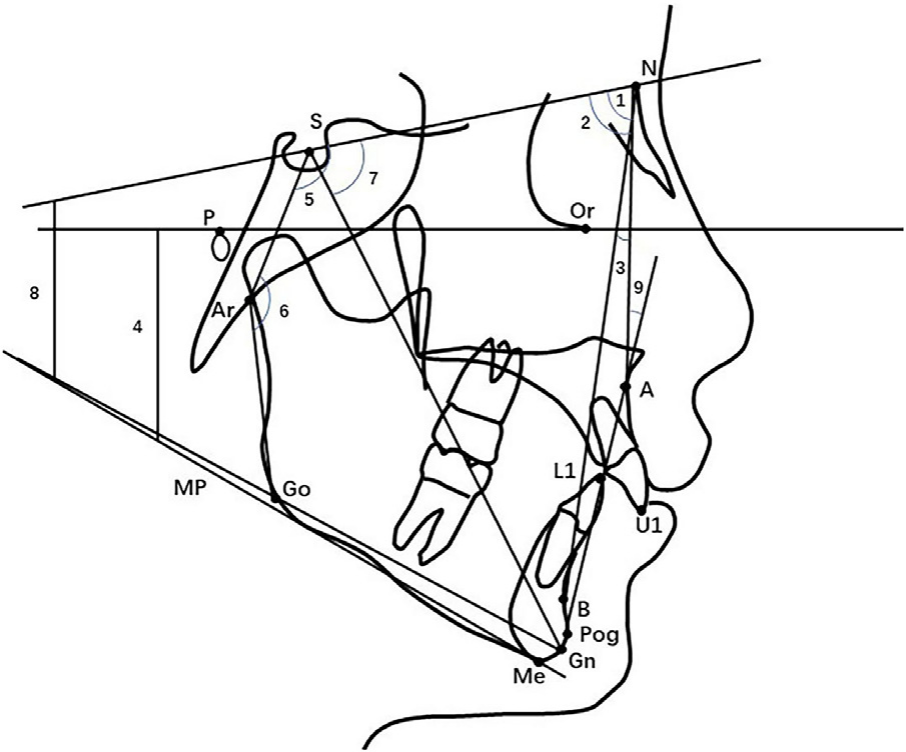
Cephalometric variables for sagittal and vertical evaluation. (1. SNA; 2. SNB; 3. ANB; 4. MP-FH; 5. N-S-Ar; 6. S-Ar-Go; 7. Y axis 8. GoGn-SN; 9. NA-PogA).

**Figure 4:**
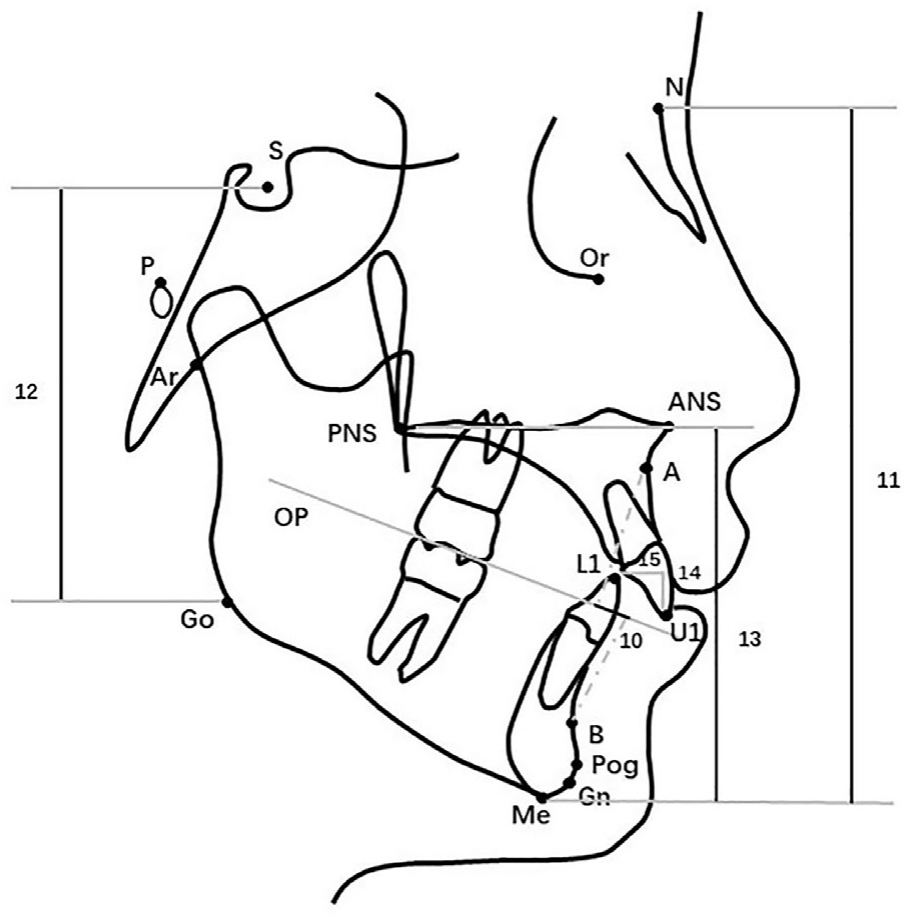
Linear cephalometric variables for sagittal and vertical evaluation. (10. Wits; 11. N-Me; 12. S-Go; 13. ANS-Me; 14. Overbite; 15. Overjet).

Lateral cephalometric radiographs and dental models were collected at the initial assessment (T0) and at a minimum of one-year follow-up (T1) for measurement. Cephalometrics were established by Dolphin Version 11.9 (Dolphin Digital Imaging, Chatsworth, USA) and dental digital models were compared in order to evaluate arch changes. The landmarks and measurements used are shown in Figure 3, Figure 4. One month later, records from each group were randomly chosen and re-evaluated by the same researcher.

### Statistical analysis

Statistical analyses were conducted utilizing SPSS software version 26.0 (IBM Corp., Armonk, USA), with a significance threshold set up at *P* < .05. The Shapiro-Wilk test was applied to assess the standard of measurements at T0 and T1. Within-group comparisons of indicators before and post-treatment were conducted via paired *t*-tests, while inter-group comparisons were performed with ANOVA.

## Results

The baseline data of the final samples for the First group and Splint group were shown in Table 1. The χ2 tests indicated no meaningful differences in the distribution of gender, CVM stage, and Angle's classification within the First group and the Splint group (*P* > .05). Furthermore, all observed variables were comparable among groups at T0, with no statistically significant differences (Table S1). Both First and Splint are effective in achieving maxillary arch expansion during the mixed dentition stage (Table S2).

### Cephalometrics effects within the groups

In the First group (Table 2), N-Me, S-Go and ANS-Me increased significantly after treatment (*P* < .05), whereas overjet and overbite decreased (*P* < .05). Besides, the ANB and NA-PogA showed no significant differences within the First group. In the Splint group (Table 2), ANB and NA-PogA decreased significantly, while SNB, N-Me, S-Go and ANS-Me increased (*P* < .05). The overjet and overbite exhibited no significant differences within the Splint group. In the Natural group (Table 2), there was a statistically increase in N-Me, S-Go and ANS-Me (*P* < .05). The ANB, NA-PogA, overjet and overbite demonstrated no significant differences within the Natural group. The SNA, MP-FH, GoGn-SN and S-Go/N-Me showed no meaningful differences within each of the three groups. As shown in Table S3, no significant differences were observed in N-S-Ar, S–Ar-Go, Y-axis, and Wits within each of the three groups.

**Table 2:** Pre- and post-treatment comparisons of cephalometric changes in the First group, Splint group, and Natural group.

	First group			Splint group			Natural group		
	T0	T1		T0	T1		T0	T1	
	Mean ± SD	Mean ± SD	*P*	Mean ± SD	Mean ± SD	*P*	Mean ± SD	Mean ± SD	*P*
SNA,	79.88 ± 2.71	79.79 ± 2.70	.854	80.84 ± 2.62	81.13 ± 2.37	.523	81.17 ± 3.72	81.62 ± 3.43	.270
SNB,	74.48 ± 2.89	74.65 ± 2.56	.654	75.56 ± 3.19	76.47 ± 3.15	.026*	75.56 ± 3.20	76.18 ± 2.78	.085
ANB,	5.41 ± 1.86	5.14 ± 1.88	.338	5.31 ± 1.83	4.61 ± 1.79	.000*	5.60 ± 1.77	5.45 ± 2.04	.376
MP-FH,	31.49 ± 4.94	31.90 ± 4.71	.480	30.78 ± 5.45	30.87 ± 6.00	.904	29.25 ± 5.04	29.00 ± 6.21	.667
GoGn-SN,	38.89 ± 5.12	39.09 ± 4.88	.763	37.29 ± 6.45	36.74 ± 6.74	.294	35.60 ± 5.21	35.04 ± 6.08	.353
NA-PogA,	12.29 ± 3.99	11.15 ± 4.27	.097	11.56 ± 4.86	9.99 ± 4.45	.001⁎⁎	12.17 ± 4.09	11.98 ± 5.27	.718
N-Me, mm	101.58 ± 4.91	105.51 ± 5.39	.000⁎⁎⁎	102.15 ± 5.40	104.79 ± 6.03	.000⁎⁎⁎	99.80 ± 5.73	102.22 ± 6.04	.000⁎⁎⁎
S-Go, mm	62.60 ± 5.33	65.41 ± 5.24	.000⁎⁎⁎	64.64 ± 4.96	67.20 ± 4.87	.000⁎⁎⁎	64.37 ± 5.33	66.58 ± 5.99	.000⁎⁎⁎
ANS-Me, mm	57.34 ± 3.26	58.98 ± 3.64	.000⁎⁎⁎	57.33 ± 4.25	58.69 ± 4.56	.000⁎⁎⁎	55.48 ± 4.06	56.95 ± 4.11	.000⁎⁎⁎
S-Go/N-Me	0.62 ± 0.04	0.62 ± 0.04	.519	0.63 ± 0.05	0.64 ± 0.05	.127	0.65 ± 0.05	0.65 ± 0.05	.117
Overbite, mm	2.65 ± 1.75	1.81 ± 1.43	.003⁎⁎	2.08 ± 1.68	2.48 ± 1.35	.271	2.11 ± 1.84	2.32 ± 1.71	.346
Overjet, mm	4.38 ± 2.40	3.51 ± 2.11	.004⁎⁎	4.79 ± 2.90	5.23 ± 2.80	.153	3.20 ± 2.19	3.39 ± 2.22	.322

T0, pre-treatment; T1, one year after treatment; First, Invisalign First; Splint, acrylic splint expander; Natural, natural growth.

⁎ *P* < .05.

⁎⁎ *P* < .01.

⁎⁎⁎ *P* < .001.

### Cephalometrics effects between the groups

#### Comparison of First vs Splint groups

Overjet and overbite decreased further in the First group when compared to the Splint group (*P* < .05) (Table 3). The SNA, SNB, ANB, MP-FH, GoGn-SN, NA-PogA, N-Me, S-Go, ANS-Me and S-Go/N-Me showed no notable differences within the First and Splint groups.

**Table 3:** Comparisons of cephalometric changes between the First group and the Splint group.

Variables	First group		Splint group		*P*
	Mean	SD	Mean	SD	
SNA,	−0.09	2.04	0.29	1.99	.544
SNB,	0.18	1.72	0.92	1.70	.125
ANB,	−0.27	1.23	−0.70	0.72	.449
MP-FH,	0.41	2.51	0.09	3.11	.715
GoGn-SN,	0.20	2.92	−0.55	2.26	.370
NA-PogA,	−1.14	2.91	−1.57	1.81	.567
N-Me, mm	3.93	2.46	2.64	2.33	.760
S-Go, mm	2.81	2.24	2.57	2.31	.730
ANS-Me, mm	1.64	1.60	1.36	1.83	.574
S-Go/N-Me	0.00	0.02	0.01	0.02	.819
Overbite, mm	−0.84	1.07	0.40	1.56	.003⁎⁎
Overjet, mm	−0.87	1.16	0.44	1.32	.001⁎⁎

First, Invisalign First; Splint, acrylic splint expander.

* *P* < .05.

⁎⁎ *P* < .01. ^⁎⁎⁎^*P* < .001.

#### Comparison of First vs Natural groups

The reduction in overjet and overbite was greater in the First group compared to the Natural group. (*P* < .05) (Table 4). There were no significant differences detected in SNA, SNB, ANB, MP-FH, GoGn-SN, NA-PogA, N-Me, S-Go, ANS-Me and S-Go/N-Me between the First and Natural groups.

**Table 4:** Comparisons of cephalometric changes between the First group and the Natural group.

Variables	First group		Natural group		*P*
	Mean	SD	Mean	SD	
SNA,	−0.09	2.04	0.65	1.94	.660
SNB,	0.18	1.72	0.89	1.84	.394
ANB,	−0.27	1.23	−0.22	−0.82	.978
MP-FH,	0.41	2.51	−0.28	−2.68	.698
GoGn-SN,	0.20	2.92	−0.45	−2.64	.364
NA-PogA,	−1.14	2.91	−0.19	−2.32	.216
N-Me, mm	3.93	2.46	2.28	1.95	.118
S-Go, mm	2.81	2.24	1.91	1.52	.393
ANS-Me, mm	1.64	1.60	1.45	1.19	.739
S-Go/N-Me	0.00	0.02	0.00	0.02	.964
Overbite, mm	−0.84	1.07	0.25	1.01	.010*
Overjet, mm	−0.87	1.16	0.22	0.88	.005⁎⁎

First, Invisalign First; Natural, natural growth.

⁎ *P* < .05.

⁎⁎ *P* < .01. ^⁎⁎⁎^*P* < .001.

#### Comparison of Splint vs Natural groups

No significant differences were found in SNA, SNB, ANB, MP-FH, GoGn-SN, NA-PogA, N-Me, S-Go, ANS-Me, S-Go/N-Me, overjet and overbite between the Splint and Natural groups (Table 5). The SNA, SNB, MP-FH, GoGn-SN and S-Go/N-Me showed no meaningful differences among the three groups (Table S4).

**Table 5:** Comparisons of cephalometric changes between the Splint group and the Natural group

Variables	Splint group		Natural group		*P*
	Mean	SD	Mean	SD	
SNA,	0.29	1.99	0.51	1.80	.963
SNB,	0.92	1.70	0.63	1.54	.489
ANB,	−0.70	0.72	−0.16	0.77	.070
MP-FH,	0.09	3.11	−0.28	−2.68	.698
GoGn-SN,	−0.55	2.26	−0.45	−2.64	.990
NA-PogA,	−1.57	1.81	−0.19	−2.32	.073
N-Me, mm	2.64	2.33	2.28	1.95	.760
S-Go, mm	2.57	2.31	1.91	1.52	.610
ANS-Me, mm	1.36	1.83	1.45	1.19	.739
S-Go/N-Me	0.01	0.02	0.00	0.02	.962
Overbite, mm	0.40	1.56	0.25	1.01	.647
Overjet, mm	0.44	1.32	0.22	0.88	.496

Splint, acrylic splint expander; Natural, natural growth.

* *P* < .05. ^⁎⁎^*P* < .01. ^⁎⁎⁎^*P* < .001.

## Discussion

As a relatively new aesthetic-oriented treatment modality, Invisalign First has been shown to effectively increase transverse width, primarily through dentoalveolar expansion.^15^ Aesthetic considerations are highly valued and may influence the selection of orthodontic appliances.^25^ However, it remains uncertain whether First can achieve growth-related sagittal and vertical skeletal effects comparable to those observed with conventional treatment acrylic splint expanders. Thus, our study aims to assess and contrast the sagittal and vertical effects of First and Splint in mixed dentition patients with MTD.

Since the sample population of this study was in the mixed dentition stage, the research results may be influenced by growth and developmental factors. Previous study also demonstrated the trend of increasing SNA and SNB angles but decreasing ANB angle in children from the transitional dentition stage to early permanent dentition.^26^ To exclude, the influence of growth during the study period, a natural growth group was included. After a year of observation, the natural growth group displayed decreasing MP-FH, increasing SNA and SNB angles, and decreasing ANB angle, which was in line with the findings of the previous study.^26^ However, these changes were not statistically significant over the one-year observation period. In contrast, significant changes were observed in the Splint group, indicating that the observed effects were attributable to the skeletal impact of splint expansion rather than natural growth.

Orthodontic outcomes may be influenced by clinician experience and operative technique. To minimize operator-related bias, all treatments were conducted at a single centre under the supervision of the same experienced senior orthodontist, using standardized diagnostic criteria and treatment protocols. Consistency in appliance placement, treatment planning, and follow-up was ensured; meanwhile, cases with inadequate compliance were excluded. These measures limited procedural and cooperation-related variability, although some inherent operator variation is unavoidable in clinical practice.

Moreover, PSM has been widely applied in contemporary scientific research to enhance experimental reliability by equalizing baseline characteristics across groups.^27^^,^^28^ Given the importance of growth-related indicators such as CVM in accounting for developmental variability, baseline differences in CVM were controlled using PSM. In addition, this application of PSM balanced other key confounding variables, including sex and Angle's classification, thereby substantially improving the comparability between the two treatment groups.

### Sagittal changes

A statistically notable reduction in ANB was found following Splint treatment, which was attributed to the increase in SNB. Numerous studies on splint therapy have reported that treatment may influence the sagittal position of the mandible.^11^^,^^29^ In our study, a mean increment of 0.92° was detected in SNB in the Splint group. As reported by Farronato et al,^8^ mandibular forward movement may occur following Splint therapy. Lima et al also reported that rapid palatal expansion was associated with forward mandibular movement, contributing to the correction of Class II malocclusion.^30^ In Splint group, a significant decrease of NA-PogA was found throughout the study period, demonstrating that Splint could improve convexity after one year of treatment, which is consistent with the findings from the literature.^31^ These findings indicate that Splint has a significant skeletal impact in the sagittal dimension. However, no statistically significant differences in sagittal indicators were detected in the First group, which may reflect the predominantly dentoalveolar nature of expansion achieved with First. Accordingly, this biological characteristic should be considered when interpreting sagittal and vertical outcomes.

Furthermore, the results showed that overjet reduced significantly in the First group. Additionally, the reduction in overbite in the First group was greater than that observed in the Natural and Splint groups, indicating a superior occlusal adjustment capability of clear aligners. Owing to ethical considerations regarding radiation exposure, CBCT imaging was not performed in the present study, precluding definitive differentiation of skeletal versus dentoalveolar contributions to sagittal changes. However, existing evidence suggests that clear aligners primarily induce dentoalveolar effects-such as posterior tooth tipping and alveolar bone remodelling-rather than midpalatal suture separation.^32^ The observed advantage in arch-form control in the First group may be attributed to the design and clinical application of clear aligners in anterior maxillary alignment, in contrast to the acrylic splint expander.

### Vertical changes

As in the vertical plane, ANS-Me, N-Me and S-Go elevated in all three groups, indicating a general tendency toward increased facial height and downward mandibular growth during this developmental period. Garib et al demonstrated that Splint increases lower anterior facial height and tilts the mandibular plane.^31^ Overcorrection was also observed when the lingual cusps of maxillary teeth was in contact with the buccal cusps of mandibular teeth, resulting in vertical increments.^33^ Recent studies have further demonstrated that Splint can generate notable stress distribution at the midpalatal suture, suggesting the potential for skeletal and vertical responses.^34^

In the present study, a slight increase in the mandibular plane angle (GoGn–SN) was observed in both the First and Splint groups, but this change did not reach statistical significance, which is consistent with findings reported by Torbaty et al in mixed dentition patients.^16^ No statistically significant intergroup differences were detected, and therefore a definitive effect of either Invisalign First or acrylic splint therapy on vertical growth or mandibular rotation cannot be established. Although previous studies have suggested that splint appliances may be considered in patients for whom vertical dimension management is a clinical concern, such recommendations are largely based on appliance design considerations, as the acrylic occlusal coverage has been hypothesized to function as a bite-blocking mechanism by limiting posterior tooth eruption.^35^^,^^36^ Given the non-significant between-group differences and confidence intervals spanning small effects in the current study, any true differences in vertical growth modulation, if present, are likely to be modest in magnitude.

Besides, overbite was found to be significantly reduced after First treatment, and it decreased greater in the First group than in the Splint group. This effect was predominantly dentoalveolar in nature, achieved through anterior tooth alignment within the expansion space and partial levelling of the curve of Spee, rather than through skeletal vertical and sagittal changes. Invisalign First may be suitable for mild transverse discrepancies and anterior alignment in mixed dentition, while acrylic splint expanders may be more appropriate in cases requiring confirmed skeletal expansion and sagittal or vertical modification. Recently, Invisalign palatal expander has been introduced, with cases demonstrating its potential to open the midpalatal suture. However, high-quality clinical studies confirming its true orthopaedic effects and long-term outcomes are still lacking, and further extensive research is needed.

### Limitation

Nevertheless, this study has certain limitations. First, confirmation of the present findings in larger cohorts is warranted, given the modest sample size after PSM and the potential risk of Type II error. Second, individual variability in craniofacial growth and patient behaviour cannot be fully controlled in clinical studies involving growing populations. Finally, a one-year follow-up period may be insufficient to capture long-term sagittal and vertical skeletal adaptations, and extended longitudinal studies are needed to comprehensively evaluate these effects. Incorporating CBCT-based analysis to assess midpalatal suture opening and the extent of posterior tooth inclination could provide further insight into the skeletal expansion effects.

## Conclusions

Splint therapy improved skeletal relationships, resulting in reduced facial convexity. First therapy produced a pronounced reduction in overjet and overbite primarily through dentoalveolar effects.

## Data availability

Data underlying the findings of this study can be obtained from the corresponding author upon reasonable request.

## Ethics approval and consent to participate

This prospective study was approved by the Ethical Committee of the Hunan Xiangya Stomatological Hospital Central South University (20200088), and informed consent was obtained from all subjects and their legal guardian(s). The study was conducted in accordance with the tenets of the 1975 Declaration of Helsinki, as revised in 2013.

## Author contributions

*Formal analysis:* Chengri Li. *Funding acquisition:* Yanqin Lu and Lingling Zhang. *Investigation:* Lanxin Lu and Lei Lei. *Methodology:* Yanqin Lu and Lingling Zhang. *Project administration:* Yanqin Lu. *Supervision:* Yanqin Lu and Lingling Zhang. *Writing–original draft:* Lanxin Lu and Lingling Zhang. *Writing–review & editing:* Lanxin Lu and Lingling Zhang.

## Funding

This research received support by the National Natural Science Foundation of China (82201083), Postdoctoral Research Foundation of China (2023M743945), Postdoctoral Fellowship Program of CPSF (GZC20233163), the Natural Science Foundation of Hunan Province (2024JJ5507, 2024JJ7625), and the Fundamental Research Funds for the Central Universities of Central South University (2023ZZTS0872).

## Clinical Trial Registration

This prospective study was registered on Chinese Clinical Trial Registry (01/02/2022, registration number: ChiCTR2200056220).

## Conflict of interest

None disclosed.

## Appendix Supplementary materials

Image, application 1
