# Supplementary material for: Sagittal and Vertical Changes After Aligner Versus Acrylic Splint Expansion in Mixed Dentition: A Prospective Trial
Source: Int Dent J. 2026 Apr 27;76(4):109579. doi: 10.1016/j.identj.2026.109579 (PMC13137170; doi:10.1016/j.identj.2026.109579)
Supplement: Supplementary file 1 [file mmc1.docx]

**Title page**

**Sagittal and vertical changes after aligner vs acrylic splint expansion in mixed dentition: A prospective trial**

Lanxin Lu^a,†^, Lingling Zhang^a,b, †^ ,Chengri Li^a^, Lei Lei^a^, and Yanqin Lu^a,*^

^a^ Department of Orthodontics, Xiangya Stomatological Hospital and Xiangya School of Stomatology, Central South University; Hunan Engineering Research Center for Digital Intelligence and Personalized Medicine; Hunan Key Laboratory of Oral Health Research, Changsha, China

^b^ Department of Dermatology & National Engineering Research Center of Personalized Diagnostic and Therapeutic Technology, Xiangya Hospital, Central South University, Changsha, China

^†^ Lanxin Lu and Lingling Zhang contributed equally to this article as co-first authors.

* Corresponding Author: Yanqin Lu, Professor and Department Chair of Orthodontics, Hunan Engineering Research Center for Oral Digital Intelligence and Personalized Medicine & Hunan Key Laboratory of Oral Health Research & Xiangya Stomatological Hospital and Xiangya School of Stomatology, Central South University, 72 Xiangya Road, 410008, Changsha, Hunan, China (E-mail: [213031@csu.edu.cn](mailto:213031@csu.edu.cn))

**Table S1** Intergroup Comparison of Baseline (T0) Cephalometric Measurements (Mean±SD)

|  | First group | Splint group | Natural group | *P* |
| --- | --- | --- | --- | --- |
| SNA, ° | 79.88±2.71 | 80.84±2.62 | 81.17±3.72 | 0.39 |
| SNB, ° | 74.48±2.89 | 75.56±3.19 | 75.56±3.20 | 0.45 |
| ANB, ° | 5.41±1.86 | 5.31±1.83 | 5.60±1.77 | 0.86 |
| MP-FH, ° | 31.49±4.94 | 30.78±5.45 | 29.25±5.04 | 0.38 |
| GoGn-SN, ° | 38.89±5.12 | 37.29±6.45 | 35.60±5.21 | 0.19 |
| NA-PogA, ° | 12.29±3.99 | 11.56±4.86 | 12.17±4.09 | 0.85 |
| N-Me, mm | 101.58±4.91 | 102.15±5.40 | 99.80±5.73 | 0.36 |
| S-Go, mm | 62.60±5.33 | 64.64±4.96 | 64.37±5.33 | 0.41 |
| ANS-Me, mm | 57.34±3.26 | 57.33±4.25 | 55.48±4.06 | 0.20 |
| S-Go/N-Me | 0.62±0.04 | 0.63±0.05 | 0.65±0.05 | 0.14 |
| Overbite, mm | 2.65±1.75 | 2.08±1.68 | 2.11±1.84 | 0.47 |
| Overjet, mm | 4.38±2.40 | 4.79±2.90 | 3.20±2.19 | 0.11 |

^*^*p* < .05; T0, pre-treatment

**Table S2** Pre- and post-treatment comparisons of arch dimensional changes in the First group, Splint group, and Natural group

|  | First group | | |  | Splint group | | |  | Natural group | | |
| --- | --- | --- | --- | --- | --- | --- | --- | --- | --- | --- | --- |
|  | T0 | T1 |  |  | T0 | T1 |  |  | T0 | T1 |  |
|  | Mean±SD | Mean±SD | *p* |  | Mean±SD | Mean±SD | *p* |  | Mean±SD | Mean±SD | *p* |
| cusp-level intercanine width, mm | 32.77±2.06 | 35.54±2.70 | .000^***^ |  | 33.96±2.79 | 37.74±2.70 | .000^***^ |  | 33.47±2.58 | 34.49±2.40 | .001^**^ |
| cusp-level first interpremolar width, mm | 38.61±3.02 | 43.05±2.95 | .001^**^ |  | 40.59±3.39 | 45.48±3.67 | .000^***^ |  | 39.62±2.42 | 40.34±1.91 | .038^*^ |
| cusp-level second interdeciduous molar, mm | 43.81±1.45 | 47.97±2.84 | .000^***^ |  | 43.72±3.46 | 49.24±2.70 | .000^***^ |  | 45.11±2.31 | 46.02±2.83 | .004^**^ |
| cusp-level first intermolar width, mm | 50.47±2.37 | 54.06±2.83 | .000^***^ |  | 50.89±2.52 | 56.55±2.95 | .000^***^ |  | 51.03±2.43 | 51.50±2.46 | .005^**^ |

^*^*p* < .05; ^**^ *p* < .01; ^***^ *p* < .001.

T0, pre-treatment; T1, one year after treatment; First, Invisalign First System; Splint, acrylic splint rapid maxillary expander; Natural, natural growth.

**Table S3** Pre- and post-treatment comparisons of cephalometric changes in the First group, Splint group, and Natural group

|  | First group | | |  | Splint group | | |  | Natural group | | | |
| --- | --- | --- | --- | --- | --- | --- | --- | --- | --- | --- | --- | --- |
|  | T0 | T1 |  |  | T0 | T1 |  |  | T0 | T1 |  |  |
|  | Mean±SD | Mean±SD | *P* |  | Mean±SD | Mean±SD | *p* |  | Mean±SD | Mean±SD | *p* |  |
| N-S-Ar, ° | 121.23±5.35 | 121.80±4.81 | .391 |  | 122.23±3.98 | 122.62±3.49 | .578 |  | 121.53±6.03 | 120.74±4.88 | .340 |  |
| S–Ar-Go, ° | 152.33±7.00 | 150.58±7.13 | .283 |  | 150.49±3.87 | 149.12±3.10 | .102 |  | 150.95±5.48 | 150.78±5.69 | .845 |  |
| Y axis, ° | 64.96±3.04 | 65.52±2.44 | .205 |  | 65.37±2.89 | 65.74±2.68 | .506 |  | 64.49±2.73 | 64.67±2.60 | .648 |  |
| Wits, mm | 0.32±3.75 | -0.21±2.74 | .303 |  | 0.19±2.88 | 0.52±2.66 | .297 |  | -0.69±3.02 | -0.44±2.94 | .537 |  |

^*^*p* < .05; T0, pre-treatment; T1, one year after treatment; First, Invisalign First System; Splint, acrylic splint rapid maxillary expander; Natural, natural growth.

**Table S4** Comparisons of cephalometric changes among the First group, Splint group and Natural group

| Variables | First group | | Splint group | | | Natural group | | Comparisons (*p*) | | |
| --- | --- | --- | --- | --- | --- | --- | --- | --- | --- | --- |
|  | Mean | SD | | Mean | SD | Mean | SD | First vs. Splint | First vs. Natural | Splint vs. Natural |
| N-S-Ar, ° | 0.57 | 2.90 | | 0.40 | 3.12 | -0.92 | -3.69 | .865 | .187 | .249 |
| S–Ar-Go, ° | -1.75 | 7.08 | | -1.37 | 3.57 | -0.01 | -3.88 | .995 | .763 | .669 |
| Y axis, ° | 0.56 | 1.91 | | 0.38 | 2.47 | 0.22 | 1.77 | .778 | .562 | .766 |
| Wits, mm | -0.53 | 2.22 | | 0.34 | 1.40 | 0.36 | 1.80 | .145 | .185 | .891 |

^*^*p* < .05; First, Invisalign First System; Splint, acrylic splint rapid maxillary expander; Natural, natural growth.


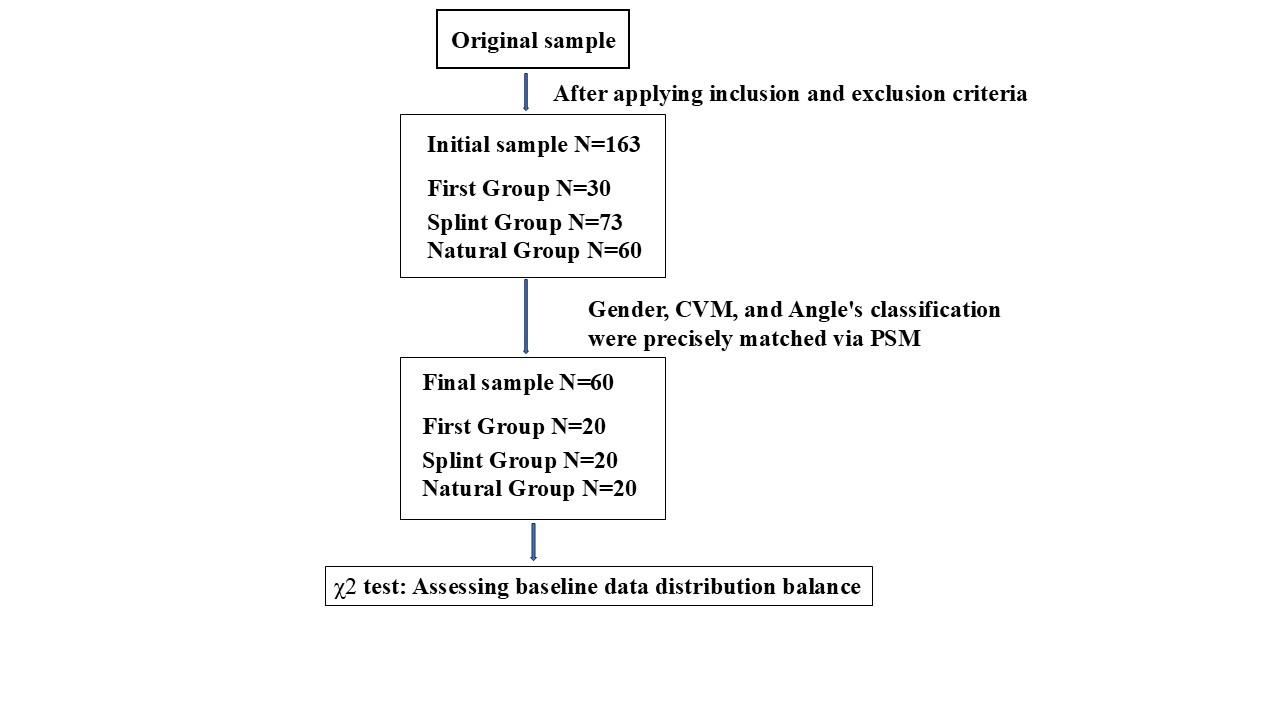


**Fig S1** Sample flow chart
